# Effect and Tolerability of Immunotherapy in Patients with NSCLC with or without Brain Metastasis

**DOI:** 10.3390/cancers14071682

**Published:** 2022-03-25

**Authors:** Birgitte Bjørnhart, Karin Holmskov Hansen, Jon Thor Asmussen, Trine Lembrecht Jørgensen, Jørn Herrstedt, Tine Schytte

**Affiliations:** 1 The Department of Oncology, Odense University Hospital, Sdr. Boulevard 29, 5000 Odense, Denmark; karin.holmskov@rsyd.dk (K.H.H.); tine.schytte@rsyd.dk (T.S.); 2 Department of Clinical Research, University of Southern Denmark, J.B. Winsløvs Vej 19,3, 5000 Odense, Denmark; 3 OPEN, Odense Patient Data Explorative Network, Odense University Hospital, J.B. Winsløws Vej 9a, 5000 Odense, Denmark; 4 The Academy of Geriatric Cancer Research (AgeCare), Odense University Hospital, Sdr. Boulevard 29, 5000 Odense, Denmark; trine.joergensen@rsyd.dk (T.L.J.); jherr@regionsjaelland.dk (J.H.); 5 The Department of Radiology, Odense University Hospital, J.B. Winsløwsvej 4, 5000 Odense, Denmark; jon.asmussen@rsyd.dk; 6 The Department of Clinical Oncology and Palliative Care, Zealand University Hospital Roskilde, Sygehusvej 10, 4000 Roskilde, Denmark

**Keywords:** non-small cell lung cancer, immunotherapy, PD-1/PD-L1 inhibition, brain metastasis, quality of life, clinical trial, magnetic resonance imaging, prospective study, screening, survival

## Abstract

**Simple Summary:**

Immune checkpoint inhibitors (ICIs) are increasingly used in the treatment of non-small cell lung cancer (NSCLC). Most randomized clinical trials have excluded patients with brain metastasis (BM), and real-life patients with NSCLC who receive ICIs are not routinely scanned with magnetic resonance imaging (MR-C) of the brain prior to ICI. This means that there are no prospective data available on the prevalence of BM or on the rate of intracranial response (ICR) attributable to ICIs. To evaluate this along with the impact of BM on quality of life and overall survival, we used MR-C as a screening tool in 159 ICI-eligible patients with advanced NSCLC prior to first ICI. At the time of ICI initiation, 28% of patients had BM. Of those who received ICI without additional early local radiotherapy or surgery, 50% had intracranial response at their first MR-C assessment. Long-term survival of patients with BM was comparable to those without.

**Abstract:**

Sparse data exist on immune checkpoint inhibition (ICI) in NSCLC patients with brain metastasis (BM), especially for those with no local therapy (LT) (whole brain radiation therapy (WBRT), stereotactic RT (SRT) or neurosurgery) preceding ICI. Our aims were to investigate the prevalence of BM, rate of intracranial response (ICR), and survival and quality of life (QoL) in real-life patients with advanced NSCLC undergoing palliative ICI. This was a prospective non-randomized study (NCT03870464) with magnetic resonance imaging of the brain (MR-C) performed at baseline resulting in a clinical decision to administer LT or not. ICR evaluation (MR-C) at week 8–9 (mRECIST criteria) for group A (LT) and group B (untreated) was assessed. Change in QoL was assessed using EQ-5D-5L. Of 159 included patients, 45 (28%) had baseline BM. Median follow-up was 23.2 months (IQR 16.4–30.2). Of patients in group A (21) and B (16), 16/37 (43%) had symptomatic BM. ICR was 8/21, 38% (complete or partial response) for group A versus 8/16, 50% for group B. No statistical difference in median overall survival of patients with BM (group A: 12.3 (5.2-NR), group B: 20.5 months (4.9-NR)) and without (22.4 months (95% 16.2–26.3)) was obtained. Baseline QoL was comparable regardless of BM, but an improved QoL (at week 9) was found in those without BM. Patients with NSCLC and BM receiving ICI had long-term survival comparable to those without BM.

## 1. Introduction

Most patients with newly diagnosed non-small cell lung cancer (NSCLC) present with metastatic disease and are thus eligible for palliative treatment only. Approximately 40% of patients with NSCLC will develop brain metastasis (BM) [1,2,3], which significantly impairs survival [4,5], increases morbidity and reduces quality of life (QoL) [5]. Historically, patients with untreated BM have a very poor prognosis with a median overall survival of only 2 months [4]. The prevalence of BM has increased during the past decade [2], which may be explained by both improved imaging diagnostics and improved cancer therapies and treatment strategies resulting in prolonged survival in selected patients [6].

For patients with NSCLC, different approaches for obtaining intracranial disease control have evolved within the last decade with regard to both local and systemic therapies. Local therapies include stereotactic radiotherapy (SRT), whole brain radiotherapy (WBRT) and neurosurgery (alone or in combination with radiotherapy (RT)). New and important systemic therapies include the third-generation epidermal growth factor receptor (EGFR) tyrosine kinase inhibitor (TKI) osimertinib, and the next- and third-generation anaplastic lymphoma kinase (ALK) inhibitors alectinib, brigatinib and lorlatinib [7,8,9,10,11]. These agents have proven highly effective in controlling intracranial disease in small subgroups of patients with NSCLC and tumors harboring the respective targetable driver mutation/alteration [6].

Immune checkpoint inhibition (ICI) has changed the clinical treatment paradigm for patients with NSCLC especially within the last 5 years. ICI is increasingly used alone or in combination with other modalities such as chemotherapy and/or radiotherapy. Data for the subgroup of patients with BM from NSCLC are sparse, since most randomized clinical trials (RCTs) of ICI have excluded patients with BM, or only included patients with stable, previously treated, and asymptomatic BM not requiring corticosteroids (CS) [12,13,14,15,16,17,18]. The purpose of screening for BM with magnetic resonance imaging of the brain (MR-C) prior to inclusion in these trials was primarily to exclude these patients. Furthermore, RCTs comprise no data on previous local BM treatment schedules (neurosurgery, WBRT, SRT). These factors make extrapolating data from RCTs to patients with NSCLC treated in the everyday clinic difficult. 

Since baseline MR-C is not routinely performed outside clinical trials for patients with NSCLC receiving ICI with palliative intent [19], the frequency as well as impact of having BM at baseline in these real-life patients is largely unknown. Data are particularly sparse for patients with untreated BM, and those who receive local treatment for BM preceding or during the initial cycles of ICI [20]. Data on QoL for this group of patients are also lacking, although this is an important clinical parameter when evaluating outcome of therapies for patients with NSCLC [21]. 

The primary endpoint of this prospective study was to determine the prevalence of BM at the time of first ICI in a consecutive group of patients with advanced NSCLC regardless of baseline CNS symptoms. Secondary endpoints were rate and duration of intracranial response for patients with BM, overall survival (OS) and QoL.

## 2. Materials and Methods

### 2.1. Design

This was a prospective cohort study including patients with advanced NSCLC eligible for ICI. ClinicalTrials.gov identifier (NC T03870464).

### 2.2. Patients and Tumor Characteristics

Key eligibility criteria were histologically verified NSCLC, advanced stage III-IV (not eligible for therapy with curative intent; surgery and/or radiotherapy), age ≥ 18 years, clinically suitable for ICI according to Eastern Cooperative Oncology Group (ECOG) performance status (PS) 0–2, and able to follow study procedures. Patients with autoimmune disease were eligible. A maximum of 10 mg of prednisolone or equivalent corticosteroid (CS) based medication was allowed at inclusion and during the ICI course. No exclusion was made based on prior therapy. Patients were included within 1 week prior to first ICI, and the index date was the date of first ICI.

**Figure 1:**
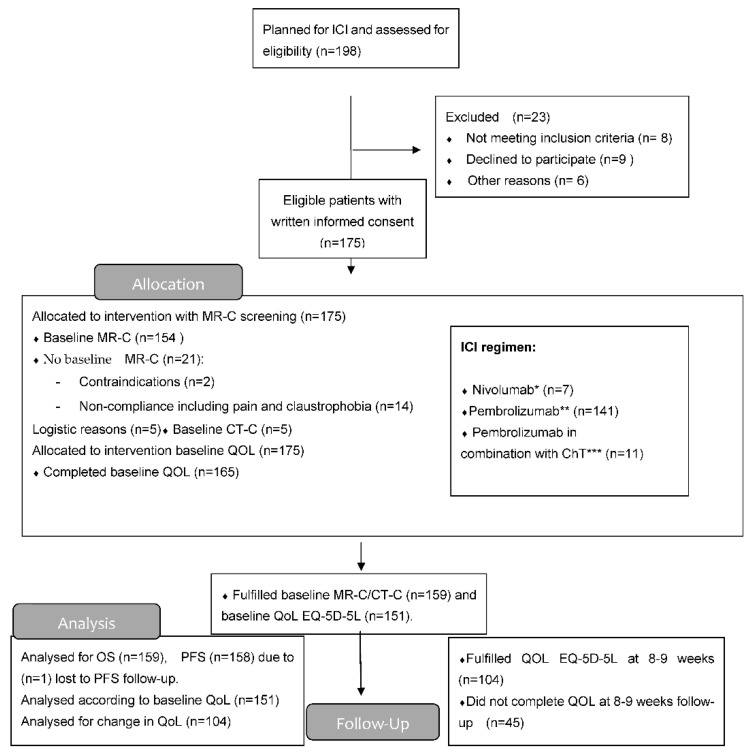
Consort flow diagram. * Dose 3 mg/kg/2nd week; ** Dose 2 mg/kg IV/3rd week; *** Dose: CT (carboplatin (AUC = 5) × (GFR mL/min + 25) IV or cisplatin 75 mg/m^2^ IV + pemetrexed (500 mg/m^2^ IV) with pembrolizumab 2 mg/kg IV. This for up to 4 cycles (every 3rd week) and after that, maintenance with same dose of pemetrexed and pembrolizumab every 3rd week for a maximum of 2 years.

**Figure 2:**
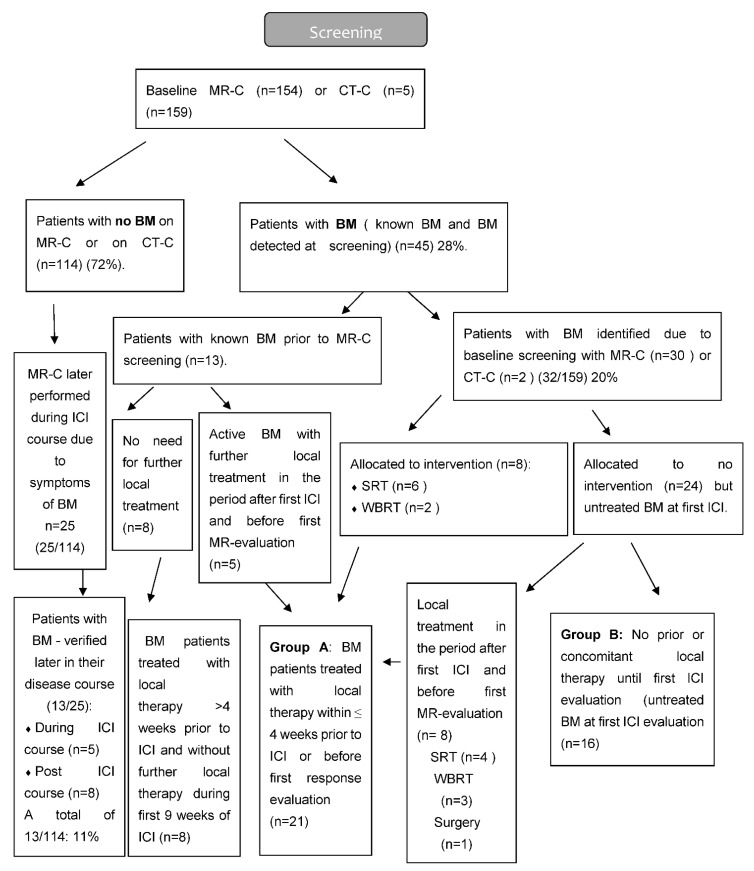
Flow diagram of MRI screening.

Based on the results of MR-C screening (for some cases, computed tomography of the brain CT-C), patients were divided into two major groups—those without BM and those with BM. The group with BM was further divided into two groups based on the addition of local therapy for BM or not. Group A: Patients with BM, who had received local therapy within the last 4 weeks prior to first ICI or during the first 6 weeks post first ICI. Group B: Patients with untreated BM, who did not receive local therapy during the first 6 weeks post first ICI. Patients with BM, who had received local therapy more than 4 weeks prior to first ICI, were included in the group with BM but neither part of group A or B (Figure 1 and Figure 2). 

A certified pathologist determined programmed death-ligand 1 (PD-L1) tumor proportion score (TPS) in extra-cranial tumor tissue by immunohistochemistry using PD-L1 IHC 22C3 pharmDx kit. EGFR and ALK fusion oncogene testing were performed in patients with non-squamous cell carcinoma at the time of primary lung cancer diagnosis, or in cases with recurrent disease through a biopsy just preceding first ICI. 

### 2.3. Treatment

Administration of ICI in both first and subsequent treatment lines was based on recommendations from the Danish Medicines Council at the particular time-point during the recruitment period [22]. For dosing schedules of nivolumab, pembrolizumab and pembrolizumab in combination with chemotherapy, see Figure 1. Local treatment of BM included neurosurgery, SRT or WBRT. The decision to offer local therapy to patients with active BM at baseline was made by the treating clinical oncologist in collaboration with the patient. If relevant, a neurosurgeon was consulted. Treatment with ICI continued until disease progression, unacceptable toxicity, or death with a maximum treatment period of 2 years (in accordance with present international treatment guidelines following palliative ICI). Treatment with ICI beyond radiologic progression was allowed in patients with clinical benefit. Local therapy for lesions in the brain or extracranially due to oligoprogression was allowed during the ICI course. 

### 2.4. Assessment

The primary endpoint was to determine the proportion of patients with BM at time of first ICI in a consecutive group of real-life patients with NSCLC and report the clinical factors characterizing this population. 

Secondary endpoints were rate- and duration of intracranial response (rICR and dICR, respectively) in those with recently treated versus untreated BM (Group A versus Group B). In addition, level of concordance between ICR and extra cranial response (ECR) was addressed. Furthermore, OS, QoL (change at 8–9 weeks compared to baseline) and PFS of patients without BM compared to patients with BM.

Relevant patient demographics were registered at the time of inclusion in a REDcap database [23]. Comorbidity according to Charlson Comorbidity Index Score (CCIS) [24], daily CS use at baseline (if any) and any CS use within 1 month prior to first ICI (yes/no)) was captured. Documentation of time-point and modality of all previously treated BM lesions were captured at baseline. Consecutive symptoms during ICI including toxicity according to the National Cancer Institute Common Toxicity Criteria version 4.0. (CTCAE) were obtained by clinical assessment and blood sampling 2–5 days prior to every ICI administration, including discontinuation due to immune related adverse events. Blood samples included standard analyses (hematological counts, liver enzymes and serum creatinine) and analyses of endocrine function.

In order to avoid delay of ICI initiation, baseline MR-C was allowed on day −30 to +7 in relation to 1st ICI. The MR-C included pre- and post-contrast T1-weighted, T2 weighted and/or T2-Fluid-attenuated inversion recovery (FLAIR) and diffusion-weighted imaging (DWI) sequences in line with the current recommendation [25]. Baseline CNS symptoms and current BM status (active or stable BM) were captured, with active BM defined as newly diagnosed, and/or non-irradiated lesions, and/or progressive lesions. Stable disease included those with previous local therapy without signs of radiologic progressive disease on baseline MR-C. Evaluation of intra- and extracranial disease was performed 8–9 weeks after first ICI and consecutively during the rest of the treatment course using MR-C and computed tomography (CT) scans, respectively. In case of SRT to the brain during the first 6 weeks after initiation of ICI, the MR-C was performed 4–5 weeks after this administration in line with pre-existing guidelines. For patients without baseline BM, later MR-C evaluation was performed only if patients developed neurological symptoms.

Response evaluation of BM was based on modified RECIST 1.1 (mRECIST) criteria allowing target lesions in the CNS of ≥5 mm (or at least twice the MR slice thickness) [26]. In case of MR-C contraindications or lack of patient compliance, a CT of the brain (CT-C) was allowed at baseline instead. According to mRECIST criteria [26], intracranial response was defined as complete response (CR) or partial response (PR). Duration of intracranial response was calculated from date of first ICI until date of first radiologically verified progression in the brain or date of death from any cause. Extracranial response was evaluated according to RECIST 1.1 using CT scan of the chest, abdomen and pelvis [27]. Progression-free survival (PFS) was calculated from date of first ICI until CT verified radiological progressive disease (PD), clinical PD leading to ICI discontinuation, PD verified by MR-C or death from any cause. OS was calculated from date of first ICI until date of death from any cause.

Evaluation of QoL was performed at baseline within 1 week prior to first ICI and at week 8–9 using the validated 5-level EQ-5D version produced by the EuroQol group (EQ-5D-5L) [28]. This is a 5-point scale reflecting: (1) No problems, (2) Slight problems, (3) Moderate problems, (4) Severe problems, and (5) Extreme problems for each of the five dimensions: mobility, self-care, usual activities, pain/discomfort, and anxiety. The score for each answer was converted into a 5-digit code describing the patients’ health state with an index value (EQ-index-score) calculated based on the crosswalk index value calculator (adjusted for use in a Danish population). This ranges from 0 (dead) to 1 (full health) [28]. 

### 2.5. Statistical Analysis

Baseline characteristics were compared using the chi-square or Fischer’s exact test for categorical variables and the unpaired *t*-test or Wilcoxon’s signed rank test for continuous variables when applicable. OS and PFS were examined using survival curves based on Kaplan–Meier (KM) estimates. Inverse KM was used to estimate median follow-up time. A Cox proportional hazards regression model was used to evaluate factors associated with OS in addition to the log-rank test. Based on univariate Cox regression analysis, a multivariate Cox regression model was constructed, including known outcome predictors such as PD-L1 expression and performance status as well as factors from the univariate analysis with a *p*-value of <0.2. Checking the proportional hazard assumption was performed using Schoenfeld residuals. For comparison of survival of patients with no BM, untreated BM and all prior treated BM, the log-rank test for trend was applied. Change from baseline to week 9 in both EQ-index-score and in EQ-VAS (which is a 0–100 scale with overall health reported by the patient on the day of questionnaire completion) was captured using a paired t-test. The index score was calculated only if patients had completed all five questions. Only patients who had completed both baseline QoL and QoL at week 8–9 were included in the analyses of EQ-VAS and EQ-index-score. Statistical analysis was performed using STATA version 17.0 with two-sided tests and a level of *p* < 0.05 was considered statistically significant [29].

### 2.6. Ethics

The study was approved by the regional research ethical committee of Southern Denmark (Project-ID: 20170155) and was conducted in accordance with the Declaration of Helsinki. All patients provided written informed consent prior to participation. The study was registered at ClinicalTrials.gov (identifier: NC T03870464). Data were deposited in a REDcap database at Odense patient explorative network (OPEN) with project number OP-521.

## 3. Results

### 3.1. Patient Characteristics

Patients were enrolled from 1 April 2018 through 31 April 2021 at the Department of Oncology, Odense University Hospital Denmark. Data cut-off was 7 September 2021. (CONSORT flow diagram, Figure 1). 

A total of 175 patients were eligible, of whom 159 patients had a baseline MR-C (*n* = 154) or CT-C (*n* = 5) obtained. Median follow-up time was 23.2 months (IQR 16.4–30.2). Two patients had EGFR mutation (2/133) and none had ALK-fusion oncogene (0/133). A total of 45 patients (28%) had BM at baseline, with the majority (*n* = 32) being identified due to screening. The vast majority had active BM (*n* = 40) compared to stable BM (*n* = 5). Neurologic symptoms were described in 18 patients with headache being the most frequent. Patients with BM were younger, more often had a histology of adenocarcinoma, and more often used CS both prior to and during the ICI course than those without BM (Table 1). BM detected by screening resulted in local treatment prior to first ICI in eight patients. Nine patients received local treatment during the first 6 weeks after initiation of ICI. The reason for local treatment after the first ICI was either delayed MR-C report (*n* = 5) or increase in clinical symptoms (*n* = 4) (Figure 2). Four patients were considered having baseline BM even though their lesions were ≤5 mm (according to visual assessment and best clinical response).

### 3.2. Efficacy

For data on all patients with BM, see Supplemental Table S1. For comparison of group A and B, see Supplemental Table S2. For patients with untreated BM (group B), intracranial response was obtained in (8/16) 50% of the evaluable patients at first MR-C evaluation with a duration of intracranial response of 16.7 months compared to 4.6 months for group A (Table 2). For those with untreated BM, a correlation of intracranial response to extracranial response was registered in 86% of cases. 

**Figure 3:**
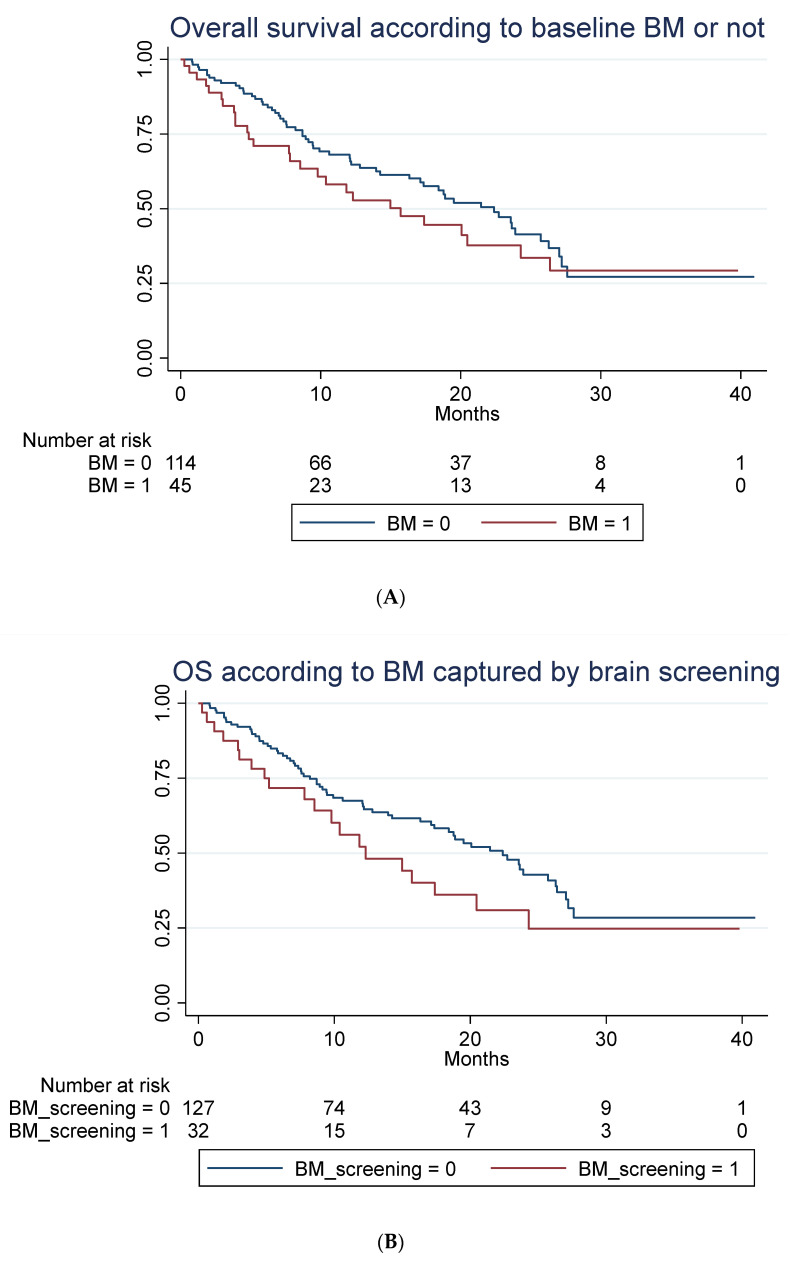

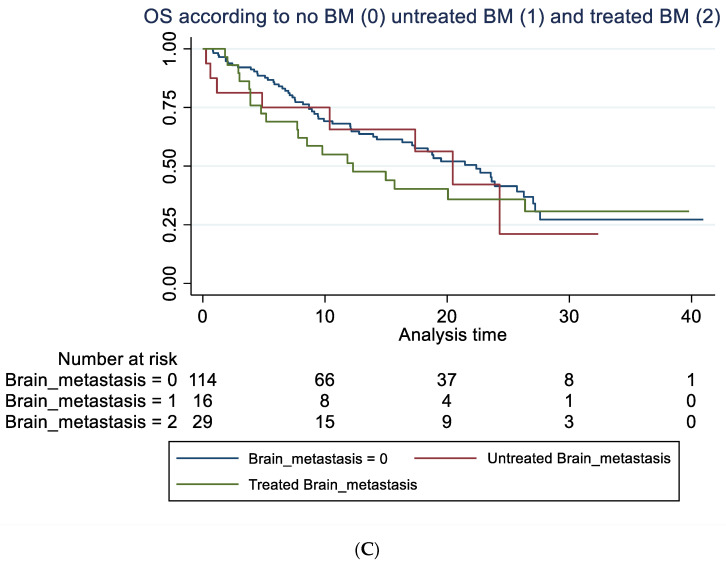
Overall survival according to brain metastasis. (**A**) Overall survival (unadjusted) in months according to baseline brain metastasis or not. Median OS of 15.7 months (95% 7.8–24.3) compared to 22.4 months (95% CI 16.2–26.3) with a hazard ratio of 1.29 (95% 0.81–2.03), *p* = 0.282. Adjusted for age, line of treatment, liver metastasis and corticosteroid use at baseline, the hazard ratio for patients with BM was 1.25 (95% 0.76–2.06), *p* = 0.381. (**B**) Overall survival (unadjusted) in months according to whether brain metastasis was detected by screening or not. Median OS of 12.3 months (95% CI 7.8–20.5) compared to 22.4 months (95% 17.1–26.3). A hazard ratio of 1.47 (95% 0.89–2.42), *p* = 0.136. Adjusted for age, line of treatment, liver metastasis and corticosteroid use at baseline, the hazard ratio for patients with BM detected due to screening was 1.45 (95% 0.87–2.42), *p* = 0.154. (**C**) Overall survival (unadjusted) in months among patients without brain metastasis (BM), with locally untreated BM, and all prior local treated BM. Hazard ratio for untreated BM was 1.21 (95% CI 0.58–2.53) and for prior treated, 1.32 (95% CI 0.78–2.22), *p* = 0.274. Adjusted for PD-L1, PS, bone metastasis and baseline corticosteroid use, the hazard ratio for untreated was 1.28 (95% 0.61–2.71), *p* = 0.511 and for prior treated, 1.14 (95% 0.66–1.98), *p* = 0.632.

A total of 85 out of 159 patients had died at time of follow-up. No statistically significant difference in OS in patients with baseline BM compared to patients without was seen, as illustrated in Figure 3A–C when adjusting for those differences in patient characteristic for those with and without BM, which might impact survival (Table 1). 

The latter illustrates OS in all patients with previously treated compared to untreated BM and those without BM. For group A, mOS was 12.3 months (95% CI: 5.2-NR) versus 20.5 (95% CI: 4.9-NR) in group B (not statistically significant). For data on PFS, rICR, ECR and dICR, see Table 2 and Table 3. For patients who obtained an early ICR (PR) more than 75% were alive for >3 years (Supplemental Figure S1). For visual inspection of ICR in two patients with untreated BM (group B) receiving ICI monotherapy, see Supplemental Figure S2. In terms of predictors of outcome, see Table 3 and Table 4 for univariate and multivariate Cox regression analysis. PD-L1 status (<50%) as well as bone metastasis were negative predictors of OS in the multivariate analysis.

### 3.3. Quality of Life

**Figure 4:**
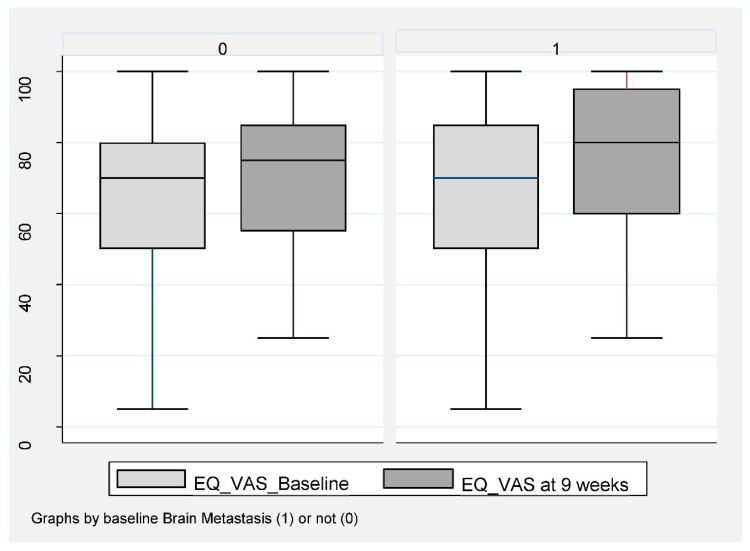
EQ-VAS in NSCLC patients at baseline compared to week 8–9 during ICI according to baseline brain metastasis or not. Box plot (illustrating median and range) of VAS-score for patients without BM (0) and with BM (1) at baseline (light gray) compared to 9 weeks (dark gray). Mean EQ-VAS score for those without BM increased at week 9 compared to baseline. Mean 65.2 (95% CI 60.4–70.0) increased to 70.7 (95% CI 66.6–74.7), *p* < 0.019. In patients with BM, a baseline mean value of 72.8 (95% CI 64.7–80.8) compared to 76.3 (95% 67.7–84.8), *p* = 0.313.

Of the 159 patients, 151 (95%) completed baseline QoL assessment and 106 (67%) at week 8–9 (Figure 1). A completed EQ-VAS for both visits was obtained for 76 patients (67%) without BM and for 28 patients (62%) with BM. For EQ-index-score, this was obtained in 71 patients (62%) without BM and 29 patients (64%) with BM. Baseline EQ-VAS and EQ-index-score were not inferior for patients with BM compared to those without. A statistically significant improvement in both EQ-VAS and EQ-index-score at 8–9 weeks compared to baseline was found in patients without BM (Figure 4). 

Patients had a mean EQ-VAS improvement from 65.2 (95% CI 60.4–70.0) to 70.7 (95% CI 88.6–74.7), *p* < 0.019 and a mean EQ-index-score improvement from 0.73 (95% 0.69–0.77) to 0.80 (95% 0.77–0.84), *p* < 0.001. No statistically significant improvement was obtained for patients with BM, with mean EQ-VAS changing from 72.8 (95% CI 64.7–80.8) compared to 76.3 (95% 67.7–84.8), *p* = 0.313 (Figure 4) and an EQ-index-score from 0.78 (95% CI 0.72–0.83) to 0.79 (95% 0.75–0.83), *p* = 0.56.

Of those with BM, Group B had the highest baseline QoL at week 8–9 (EQ-VAS at week 8–9 was 87.5), (Table 2) and the same trend towards improvement.

## 4. Discussion

To the best of our knowledge, this is the first real-life prospective study on prevalence of BM in ICI eligible patients with NSCLC providing data on QoL and intracranial response to ICI in relation to long-term OS. We reported that 28% of real-life patients with NSCLC currently eligible for ICI had BM, which was higher than the 6–17% with BM participating in the pivotal RCTs [12,13,14,15,16,17,30,31], but fully in line with the large retrospective real-life study by Hendriks et al. [32]. In the RCTs mentioned, all patients with BM had previously received local treatment for BM and had stable BM, as opposed to 89% of our population with active BM, of whom 40% had previously untreated BM. We reported intracranial response in around 50% of evaluable patients with locally untreated BM. Although some evidence of intracranial activity of ICI in patients with NSCLC already exists [33,34], our study adds further knowledge to the proof of concept, that ICI has an effect on BM. As opposed to Goldberg et al. [33,34], who (1) included patients without neurological symptoms only, (2) did not include patients requiring CS and (3) did not include patients with untreated or progressive BM > 20 mm, our study included a large proportion of patients with active and/or symptomatic BM requiring CS before and during ICI. Nor did we have exclusion criteria based on the size of BM. Furthermore we report that duration of intracranial response in selected patients with untreated BM was not inferior to that in patients with locally treated BM. Thus, our study illustrates that long-term survival (>3 years) is actually possible in patients with active BM, even in selected patients who do not receive local therapy during their early ICI course but are followed with clinical control and regular MR-C.

Current data on patients with NSCLC and BM receiving ICI come from RCTs, single arm phase I/II trials, or expanded access programs, all of which have included pre-selected patients, resulting in almost no data on patients with active and/or symptomatic BM [18,35,36]. The only present data comparable to the everyday clinical patient come from retrospective studies [32,37,38,39,40,41,42,43], and prospective data within this area are warranted [20]. In line with these retrospective studies in patients with advanced NSCLC, our study report that patients with BM are younger and more often have a histology of adenocarcinoma than those without BM [32,37]. During the last decades, adenocarcinomas have constituted a still larger proportion of all NSCLC cases. This histology is much more prone to be mutation-driven compared to squamous cell carcinomas. They have a particular high risk of intracranial spread. For those without oncogenic drivers, it still remains unclear whether the histology in itself contains other biological features predisposing these patients to growth and tumor cell migration to and within the brain.

Importantly, we report that patients with BM have a mOS, mPFS and baseline QoL level fully comparable to patients with NSCLC without BM. Patients with BM did, however, more often use CS both prior to and at the time of ICI initiation, which might reflect that these patients have different symptoms. This might need attention. The improvement in QoL at week 8–9 was only statistically significant for patients without BM, illustrating the overall early benefit of ICI in this patient group. In multivariate analysis, the well-established biomarker PD-L1 ≥50% was a predictor of better outcome, in line with the present literature. Bone metastasis, on the other hand, was only one of several metastatic sites in this study that significantly impaired OS. Most RCTs including ICI do not include baseline data on bone metastasis, but two recent nationwide Danish retrospective studies reported that bone metastasis as opposed to brain metastasis may be considered an important negative predictor of poorer survival [40,41]. Other recent studies also illustrate the complexity and heterogeneity of the NSCLC disease and the difficult interplay between sex, cancer-profile histology, and comorbidity in terms of prognosis of NSCLC [44,45]. For the current study, no significant difference in terms of OS was observed in relation to sex, PS or CCIS.

Despite the lack of consecutive MR-C in those patients who did not present with BM at baseline, we reported that 11% (13/114) of those without baseline BM developed symptomatic BM verified by MR-C later on during their disease course (not necessarily during ICI). This number added to the 28% having baseline BM (39% in total), is in alignment with the literature in general, stating that 30–50% [1,2,3] of NSCLC patients will be diagnosed with BM eventually. Furthermore, since we report that around 20% of real-life patients with NSCLC eligible for ICI haveBM not previously diagnosed, it should be considered standard to perform MR-C at baseline prior to ICI initiation in order to obtain correct TNM staging based on latest IASCLC criteria [46]. This is in alignment with the recently updated EANO–ESMO guidelines for diagnosis, treatment and follow-up of patients with BM from solid tumors [25]. MR-C at baseline is already implemented in the international treatment guidelines of patients with NSCLC harboring driver mutations /ALK translocation. Including patients with BM in TKI RCTs has lead to the use of third-generation TKIs such as osimertinib as standard therapy in first line for this subgroup of patients regardless of BM status. This is due to its superior effect in preventing BM, controlling preexisting BM disease and improving survival. Using MR-C as a screening tool at baseline in those patients with NSCLC without driver mutations/ALK translocation, who are eligible for ICI, makes it possible to identify BM early on in the ICI treatment course with the potential of offering an individualized treatment strategy. This might lower the patient’s risk of developing serious CNS symptoms later on and might improve outcome in terms of both survival and QoL. Based on our results, we have now implemented baseline MR-C screening for all patients with NSCLC prior to palliative systemic treatment in our department In order to be able to offer a multidisciplinary treatment strategy for these patients with BM, close collaboration among clinical oncologists, radiation therapists, neurosurgeons and radiologists, and in some cases among different oncologic centers, is mandatory.

Since our study illustrates that patients with NSCLC and BM might obtain durable responses leading to long-term survival without early impairment of QoL, it is important that these patients are offered ICI. Before the implementation of ICI for patients with NSCLC, long-term survival in patients with BM was rare except for those with driver mutations/alterations [47]. Importantly, the mOS in both group A and B was superior to the mOS reported from previous studies on patients with BM receiving palliative chemotherapy of around 7 months [48,49]. This adds further evidence to the importance of offering ICI to real-life patients with NSCLC and BM and not limiting treatment options to RT and chemotherapy. It also emphasizes the importance of including patients with NSCLC and BM in RCTs. In the group of patients with previously untreated BM (group B), a remarkable median duration of intracranial response of 16.7 months was obtained compared to that of 4.6 months in those with previously treated BM (group A). These numbers should be interpreted with great caution due to the small numbers in each group and the non-randomized design. However, besides the obvious difference in overall prognostic factors among groups, one could speculate whether it also reflects the use of less immunosuppressive CS in the untreated group. Corticosteroids, which are given to most patients during courses of cranial RT, might impair the efficacy of ICI and reduce the chance of obtaining a long-term response and survival with ICI [50]. Our study illustrates the importance of obtaining an early intracranial response. This is supported by the promising flattening of the survival curve for 75% of those obtaining an early intracranial PR (reaching more than 40 months of survival) and for 25% with early intracranial SD, inducing a hope of durable response in a subset of patients with BM. This, in combination with a correlation of intracranial response to extracranial response in 86% of cases for those with BM initially left untreated, suggests that ICI in combination with close MR-C control might be a potential initial treatment strategy in a subgroup of NSCLC patients with BM, in particular, for those with smaller size BM, a PD-L1 (TPS) status ≥50%, ICI given in first line and for those without neurological symptoms. Our descriptive results support the recently proposed model of treatment strategy for BM during ICI suggested by Eguren-Santamaria [51].

The limitations of this study are, among others, the small size of the study population and the lack of randomization based on the distribution of local therapy or not. By performing descriptive and exploratory comparisons among group A and B, we tried to gather clinically important and warranted data. A limitation was that these groups were not matched in terms of known prognostic factors. Probably due to clinicians’ selection bias, patients in the untreated group were more often treated with ICI in first line, and both size and number of lesions were smaller/lower. This probably also resulted in a higher baseline QoL. Data on the investigated subgroups are presented in Table 2, Supplemental Tables S1 and S2, in order to make this transparent. The inclusion of patients receiving ICI in different lines of therapy adds to the heterogeneity, and larger randomized studies should stratify for this. Additionally, patients were included based on the clinical approval of the ICI drug alone or in combination with chemotherapy, which changed during the recruitment period. This resulted in a small subgroup of patients receiving ICI in combination with chemotherapy, which is not optimal when evaluating responses to a particular drug (ICI).

The comparable OS in patients with BM compared to those without BM was in correlation with the few previous studies from malignant melanoma [33,34] and the pooled retrospective analysis of stable BM reported in the keynote studies in patients with NSCLC [49]. In our study, this finding was seen, despite the fact that most patients had active and many symptomatic and/or untreated BM during the early ICI course.

A recent RCT in patients with malignant melanoma did include patients with symptomatic BM using combination ICI therapy and reported benefits even in this subgroup, but to a lesser extent than those who were asymptomatic [52]. As in this study, future RCTs in patients with NSCLC should include patients with active and/or untreated BM as well, in order to gain more knowledge on the effect of ICI in this heterogeneous group of patients. Differences between cancer subtypes in terms of effect of ICI regimens (ICI alone, combination ICI or ICI in combination with chemotherapy) as well as RT sensitivity of the particular tumor tissue should be taken into account when designing RCTs including patients with NSCLC and BM.

## 5. Conclusions

This study illustrates that patients with advanced NSCLC and BM receiving palliative ICI have a long-term survival comparable to those without BM. MR-C screening for BM in patients with advanced NSCLC prior to palliative ICI should be considered standard in order to optimize each patient’s individual treatment course. Since intracranial response was observed in 50% of patients with locally untreated BM with a high correlation to extracranial response, initiation of ICI with close MR-C control but without early local therapy may be an option for selected subgroups with BM. Our data emphasize the importance of including patients with NSCLC and BM in RCTs, and we need large prospective studies investigating combinations of RT and ICI in real-life patients in the nearest future. This is necessary in order to gain more knowledge on how to personalize treatment for this heterogenic group of patients, thereby aiming for further improvement of OS and QoL.

## Figures and Tables

**Table 1:** Baseline patient characteristics according to baseline brain metastasis (BM) or not.

Baseline Characteristics (*n* = 159)	No Baseline Brain Metastasis (*n* = 114)	Baseline Brain Metastasis (*n* = 45)	*p*-Value
**Age**, median, range	69 (39–83)	65 (35–79)	0.025
**Sex**, *n* (%)			
Men	60 (53)	17 (38)	
Female	54 (47)	28 (62)	0.091
**Histology**, *n* (%)			
Squamous carcinoma	23 (20)	0	<0.001
Adenocarcinoma	76 (67)	41 (91)	
Carcinoma-not specified	15 (13)	4 (9)	
**Disease stage**, *n* (%)			
III	15 (13)	0	
IV	99 (87)	45 (100)	0.006
**Metastatic locations**, *n* (%)			
-Liver	7 (6)	8 (18)	0.024
-Bone	35 (31)	15 (33)	0.747
-Adrenal	19 (17)	8 (18)	0.867
**Line of treatment**, *n* (%)			
1	77 (68)	24 (53)	0.094
≥2	37 (32)	21 (47)	
**CCIS**, *n* (%)			
0–1	58 (51)	29 (64)	
≥2	56 (49)	16 (36)	0.122
**PD-L1** (*n* = 159), *n* (%)			
<50%	28 (25)	16 (36)	
≥50%	86 (75)	29 (64)	0.163
**EGFR** (*n* = 133), *n* (%)	1 (1)	1(2)	0.544
**Performance status ECOG**, *n* (%)			
0	34 (30)	12 (27)	
1	64 (56)	27 (60)	
2	16 (14)	6 (13)	0.902
**Use of CS within 1 month prior to ICI**, *n* (%)			
No	89 (78)	25 (56)	
Yes	23 (22)	20 (44)	0.005
**Use of regular CS during ICI (max dose of 10 mg/day)**, *n* (%)			
No	105 (92)	32 (42)	
Yes	9 (8)	13 (30)	0.001
**Baseline EQ-5D-5L**			
EQ VAS (*n* = 151)	*n* = 109	*n* = 42	
median, range	70 (5–100)	70 (5–100)	0.493
EQ-5D index (*n* = 150)	*n* = 107	*n* = 43	
median, range	0.770 (0.169–1.0)	0.794 (0.441–1.0)	0.221

**Table 2:** Objective response rate (ORR), intracranial response rate (ICR), overall survival (OS), progression-free survival (PFS), site of first relapse, duration of response and reason for discontinuation of ICI in patients with no BM and in BM (separate columns for group A and B). Patients with BM treated prior to 4 weeks before ICI initiation are not listed separately (*n* = 8). NA: Not applicable.

Outcome	No Brain Metastases at Baseline (*n* = 114)	Brain Metastasis at Baseline (*n* = 45)	Group A (*n* = 21)	Group B (*n* = 16)
**OS**, median, months	22.4	15.7	12.3	20.5
[95% CI]	(16.2–26.3)	(7.8–24.3)	(5.2-NR)	(4.9-NR)
**PFS**, median, months [95% CI]	7.8 (6.0–9.4)	5.2 (3.3–7.6)	5.2 (2.1–9.8)	7.6 (2.6–19.3)
**Extracranial ORR, *n* (%)**				
Complete response	2 (2)	1 (2)	1 (5)	0 (0)
Partial response	42 (37)	23 (51)	8 (38)	12 (75)
Stable disease	34 (30)	7 (16)	5 (24)	1 (6)
Progressive disease	29 (25)	11 (24)	7 (33)	1 (6)
Not evaluable	5 (4)	1 (2)	0 (0)	NA
No assessment	2 (2)	2 (4)	0 (0)	2 (13)
**Intracranial response at 8–9 weeks, *n* (%)**				
Complete response	NA	1 (2)	0 (0)	1 (6)
Partial response	NA	14 (31)	8 (38)	7 (44)
Stable disease	NA	14 (31)	5 (24)	4 (25)
Progressive disease	NA	11 (24)	7 (33)	0 (0)
Not evaluable	NA	2 (4)	0	2 (13)
No assessment	NA	3 (7)	1 (5)	2 (13)
Correlation to extracranial response, yes/no *n* (%)	NA	32/42 (76)	14/20 (70)	12/14 (86)
No assessment	NA	3 (7)	1 (5)	2 (13)
**Duration of ICR**, months, median (95%CI)	NA	5.8 (3.7–16.7)	4.6 (2.4–14.5)	16.7 (0.7-NR)
**Number of patients who progressed intracranially, including death:**				
Yes	NA	29 (64)	15 (71)	8 (50)
No	NA	15 (33)	5 (24)	8 (50)
Unknown	NA	1 (2)	1 (5)	0
**Site of first relapse:**				
-Intracranially	NA	7(16)	4 (19)	1 (6)
-Extracranially	NA	15(33)	7 (33)	7 (43)
-Both	NA	8 (18)	5 (24)	1 (6)
-No PD	NA	11 (24)	4 (19)	5 (31)
-No assessment	NA	4 (9)	1 (5)	2 (13)
**Discontinuation of ICI *n* (%) due to:**				
Progressive disease	52 (46)	24 (53)	13/21 (62)	6/16 (38)
irAE related toxicity (may be in combination with PD)	48 (42)	16(36)	6/21 (29)	7/16 (44)
Death without verified PD	3 (3)	4(9)	1/21 (5)	3/16 (19)
Declining performance status not due to PD	7 (6)	2(4)	2/21 (10)	0/16 (0)
-ICI is ongoing	11 (10)	4 (8)	1/21 (5)	2 (13)
-Patient is dead	58 (51)	27 (60)	14 (67)	8 (50)
-Patient has had PD	83 (73)	34 (76)	17 (81)	11 (69)
**Other systemic treatment after ICI**				
Chemotherapy	44 (39)	14 (31)	8 (38)	5 (31)
**QoL change from baseline to week 9:**	*n* = 76	*n* = 28	*n* = 12	*n* = 10
EQ-VAS	65–71	73–76	72–72	80–87.5
	*n* = 71	*n* = 29	*n* = 14	*n* = 10
EQ-index-score	0.73–0.80	0.78–0.79	0.75–0.74	0.78–0.84

**Table 3:** Univariate Cox proportional hazard regression analysis for OS and PFS.

Baseline Characteristics	OS HR (95% CI)	*p*-Value	PFS HR (95% CI)	*p*-Value
Age (years) (reference ≥70 vs. <70)	1.06 (0.69–1.64)	0.773	1.22 (0.84–1.77)	0.288
Performance status (2 vs. 0–1)	1.55 (0.86–2.81)	0.146	1.29 (0.77–2.16)	0.335
Sex (female vs. male)	0.88 (0.58–1.35)	0.564	1.15 (0.80–1.67)	0.442
Histology (adenocarcinoma vs. others)	0.95 (0.59–1.53)	0.827	0.98 (0.65–1.48)	0.919
Line of treatment (1st vs. ≥2)	1.19 (0.77–1.84)	0.426	1.27 (0.88–1.85)	0.203
Liver metastasis (yes vs. no)	1.27 (0.61–2.63)	0.522	1.58 (0.85–2.96)	0.151
Brain metastasis (yes vs. no)	1.29 (0.81–2.03)	0.282	1.30 (0.87–1.94)	0.198
Bone metastasis (yes vs. no)	2.14 (1.37–3.34)	0.001	1.54 (1.04–2.27)	0.031
PD-L1 expression (≥50% vs. 0–49%)	0.34 (0.21–0.53)	<0.0001	0.42 (0.28–0.63)	<0.0001
Use of corticosteroids at baseline (yes vs. no)	1.77 (1.01–3.10)	0.047	1.43 (0.84–2.45)	0.181
Use of corticosteroids within 1 month of ICI (yes vs. no)	1.67 (1.06–2.65)	0.027	1.27 (0.85–1.90)	0.248

**Table 4:** Multivariate Cox proportional hazard regression analysis for OS.

Baseline Characteristics	OS HR (95% CI)	*p*-Value
Performance status (2 vs. ≤1)	1.60 (0.93–3.07)	0.157
PD-L1 (≥50% vs. <50%)	0.37 (0.22–0.60)	<0.0001
Bone metastasis (yes vs. no)	1.70 (1.06–2.73)	0.027
Use of corticosteroids at baseline (yes vs. no)	1.48 (0.81–2.69)	0.198

## Data Availability

Data are stored in a REDcap database at OPEN with ID OP-521. Specific datasets involved in this publication might be obtained from the corresponding author by request.

## Supplementary Materials

The following supporting information can be downloaded at: www.mdpi.com/article/10.3390/cancers14071682/s1, Table S1: Patients with brain metastasis, disease and treatment characteristics. Table S2: Comparison of Group A (locally treated BM) and Group B (untreated BM). Figure S1: Overall survival according to intracranial response at week 8–9. Figure S2: Magnetic resonance imaging of the brain (MR-C) illustrating intracranial response (ICR) in two different patients (A and B) with untreated BM.
