# Supplementary material for: Effect and Tolerability of Immunotherapy in Patients with NSCLC with or without Brain Metastasis
Source: Cancers (Basel). 2022 Mar 25;14(7):1682. doi: 10.3390/cancers14071682 (PMC8997168; doi:10.3390/cancers14071682)

cancers

MDPI

Article

Effect and tolerability of immunotherapy in patients with NSCLC with or without brain metastasis.

Birgitte Bjørnhart [1,2,3,4]*, Karin Holmskov Hansen [1,3], Jon Thor Asmussen [5], Trine Lembrecht Jørgensen [4], Jørn Herrstedt [4,6] and Tine Schytte[1,2,4].

Supplemental Table 1: Patients with brain metastasis, disease and treatment characteristics n=45

	**All with BM (including prior surgery resected BM) (*n* = 45)**	**BM with local treatment prior to first ICI (*n* = 21)**	**BM without treatment prior to first ICI (*n* = 24 *)**
Size of largest BM at baseline in mm, median, (range)	11 (0-33)	11 (0-33)	10.5 (2-23)
Number of metastasis, n (%):			
0 (resected)	2 (4)	2 (10)	0
1	16 (36)	4 (19)	12 ((50)
2	9 (20)	5 (24)	4 (17)
3	4 (9)	1 (5)	3 (13)
≥4	14 (31)	9 (43)	5 (21)
Treatment prior to first ICI, *n* (%):			
Surgery	4 (9)	4(19)	NA
SRT	12 (27)	12 (57)	NA
WBRT	5 (11)	5 (24)	NA
None	24 (53)	0	24 (100)*
CNS symptoms at baseline			
No	27 (60)	9 (43)	18 (75)
Yes	18 (40)	12 (57)	6 (25)
Details on CNS symptoms (multiple answers possible):			
-dizziness	6 (13)	4 (19)	2 (8)
-headache	11 (24)	9 (43)	2 (8)
-nausea	4 (17)	3 (14)	1 (4)
-focal symptoms	6 (13)	3 (14)	3 (13)

*One patient had both prior treated BM and untreated BM but was included in the untreated group. NA: Not applicable.

Supplemental Table 2: Comparison of Group A (locally treated BM) and Group B (untreated BM).

	**Treated BM <= four weeks prior to first ICI or within the first 6 weeks after 1. ICI (*n* = 21)**	**Untreated BM prior to ICI and before first evaluation (No prior or concomitant treatment) (*n* = 16)**
Size of largest BM at baseline in mm, median, (range)	14 (0-26)	8.0 (2-23)
Number of metastasis (including resected)		
0	0	0
1	6 (29)	9 (56)
2	4 (19)	3 (19)
3	2 (10)	1 (6)
≥4	9 (43)	3 (19)
Treatment prior to first ICI, *n* (%):		
Neurosurgery	3 (14)	0
SRT	11 (52)	1* (6)
WBRT	7 (33)	0
None	0	0
CNS symptoms at baseline		
No	7 (33)	14 (88)
Yes	14 (67)	2 (13)
Status BM at baseline		
-Active	21 (100)	16 (100)
PD-L1 status extracranially		
<1	2 (10)	1 (6)
≤1>50	6 (29)	3 (19)
≥ 50	13 (62)	12 (75)
Line of ICI		
1	8 (38)	13 (81)
≥2	13 (62)	3 (19)

* One patient had both prior treated BM and untreated BM but was included in the untreated group.

Supplemental Figure 1:

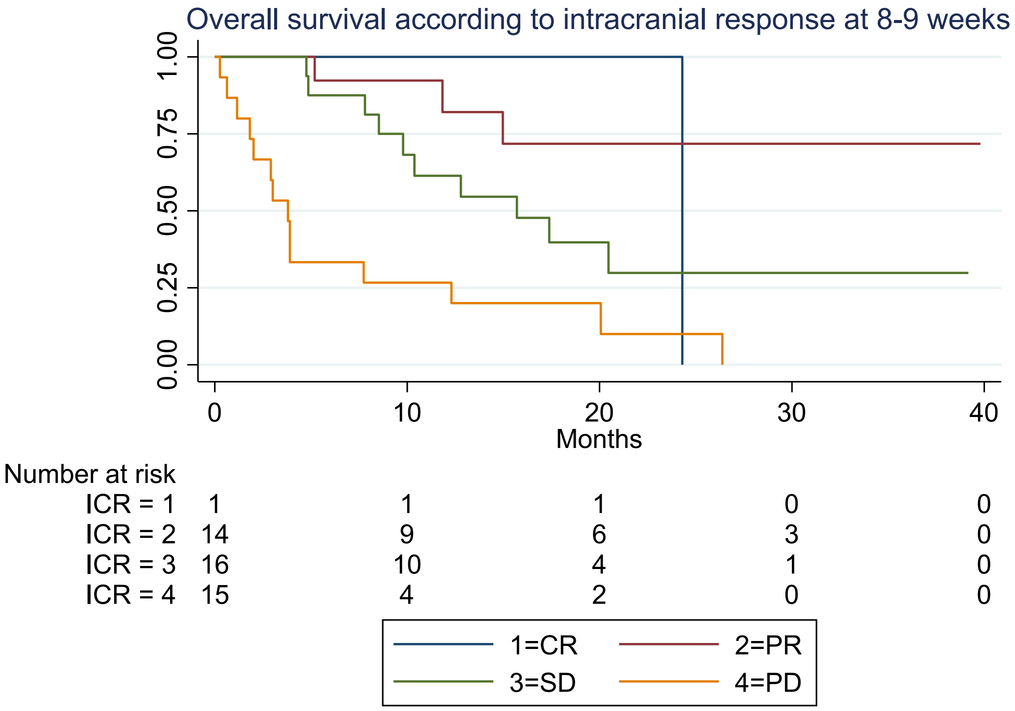

Overall survival in months in patients with brain metastasis (BM) according to intracranial response at week 8-9 (CR: Complete response, PR: Partial response, SD: Stable disease, PD: Progressive disease).

Supplemental Figure 2: Magnetic resonance imaging of the brain (MR-C) illustrating intracranial response (ICR) in two different patients (A and B) with untreated BM. Baseline MR-C (left) and at first evaluation at 8-9 weeks (right).

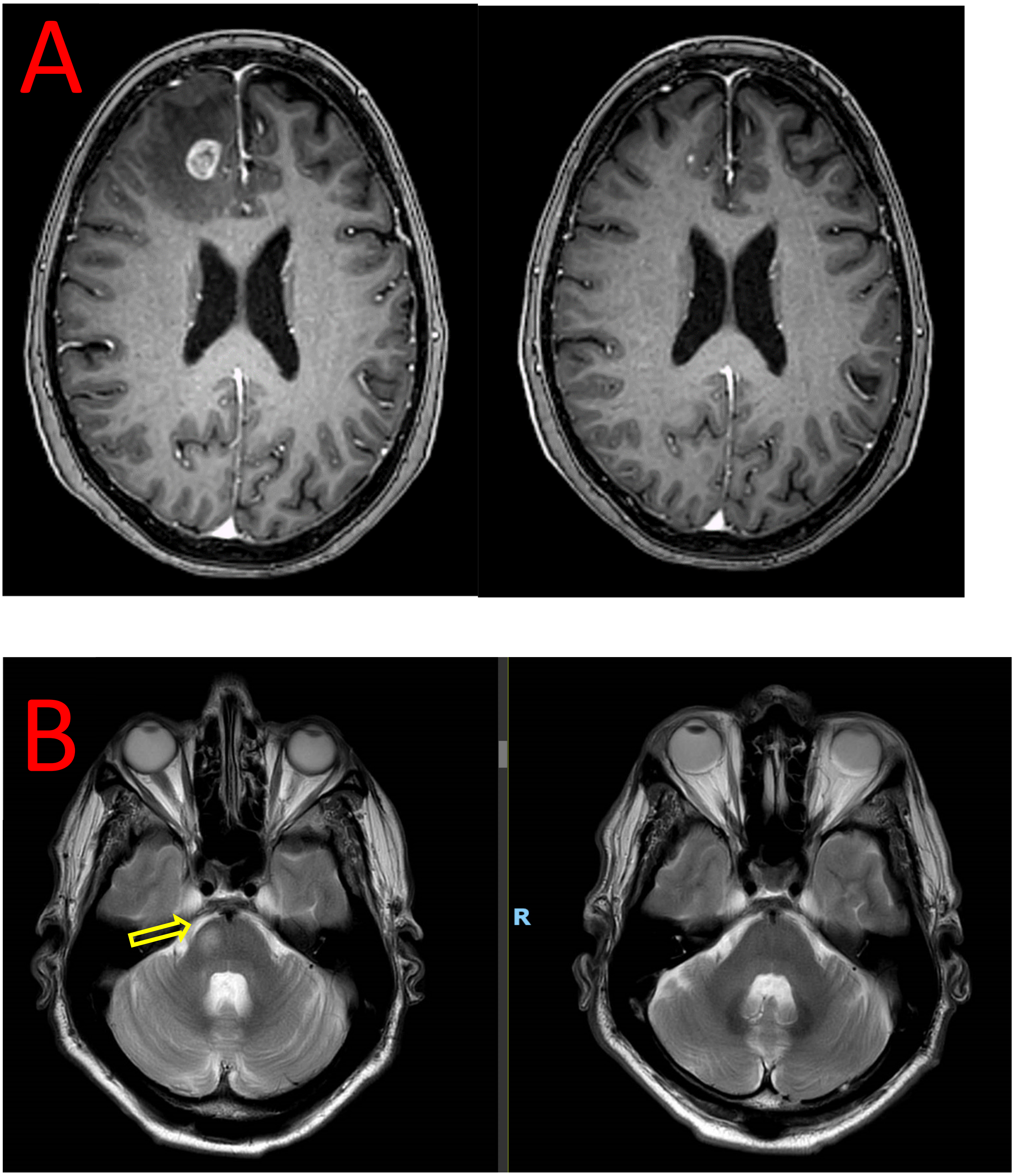

Supplement: Supplementary file 1 [file cancers-14-01682-s001.zip › cancers-1651179-SI.pdf]
